# Biomarkers for nutrient intake with focus on alternative sampling techniques

**DOI:** 10.1186/s12263-016-0527-1

**Published:** 2016-04-16

**Authors:** T. Holen, F. Norheim, T. E. Gundersen, P. Mitry, J. Linseisen, P. O. Iversen, C. A. Drevon

**Affiliations:** 1 Division of Molecular Nutrition, Department of Nutrition, Institute of Basic Medical Sciences, University of Oslo, POB 1046, Blindern, 0317 Oslo, Norway; 2 Department of Medicine/Division of Cardiology, David Geffen School of Medicine, University of California, Los Angeles, Los Angeles, CA USA; 3 Vitas AS, Olso Innovation Park, Gaustadalleen 21, N-0349 Oslo, Norway; 4 Institute of Epidemiology II, Helmholtz Zentrum München, Neuherberg, Germany

**Keywords:** Dried blood spots (DBS), Biomarkers, Lipidomics, Nutrients, Microbiome, Diet

## Abstract

Biomarkers of nutrient intake or nutrient status are important objective measures of foods/nutrients as one of the most important environmental factors people are exposed to. It is very difficult to obtain accurate data on individual food intake, and there is a large variation of nutrient composition of foods consumed in a population. Thus, it is difficult to obtain precise measures of exposure to different nutrients and thereby be able to understand the relationship between diet, health, and disease. This is the background for investing considerable resources in studying biomarkers of nutrients believed to be important in our foods. Modern technology with high sensitivity and specificity concerning many nutrient biomarkers has allowed an interesting development with analyses of very small amounts of blood or tissue material. In combination with non-professional collection of blood by finger-pricking and collection on filters or sticks, this may make collection of samples and analyses of biomarkers much more available for scientists as well as health professionals and even lay people in particular in relation to the marked trend of self-monitoring of body functions linked to mobile phone technology. Assuming standard operating procedures are used for collection, drying, transport, extraction, and analysis of samples, it turns out that many analytes of nutritional interest can be measured like metabolites, drugs, lipids, vitamins, minerals, and many types of peptides and proteins. The advantage of this alternative sampling technology is that non-professionals can collect, dry, and mail the samples; the samples can often be stored under room temperature in a dry atmosphere, requiring small amounts of blood. Another promising area is the potential relation between the microbiome and biomarkers that may be measured in feces as well as in blood.

## Background

Reliable knowledge about the relationship between food intake and nutritional status is very important for improving the quality of nutritional research. Most data generated in large epidemiological studies in humans are based on memorizing or monitoring food intake from the participants [1, 2]. These methods are inaccurate and represent challenges concerning under- as well as over-reporting of certain foods [3]. Based on these facts, there is an urgent need for biomarkers of objectively describing both intake and nutritional status. Different omics analyses can be applied on all types of tissues and biological liquids to improve dietary assessments [4]. However, the use of objective biochemical variables is complicated by confounding factors. The amount and composition of a biomarker in tissues or blood depend on multiple processes such as digestion and absorption in the gastrointestinal tract, transport in the blood, uptake, distribution, and metabolism in a variety of cells, and excretion via the kidney and gastrointestinal tract. All these processes involve multiple gene products with polymorphisms potentially creating large individual variations [5]. Moreover, different physiological states like fasting feeding, cold, warm, resting, exercising, sex, menstrual cycle, pregnancy, lactation, and age might have effects on the lipid spectrum. Finally, the nutrient composition of ingested food, endogenous production of different molecules, flux into and out of various compartments in the body, and sampling time points, must be considered when omics data are interpreted. All these considerations make it likely to suggest that the rapid development of biomarker measurements to be discussed in this review will represent an important addition to the information obtain by classical methods for registration of food intake simply because the two approaches intend to monitor different variables (food intake with all its inaccuracies) and a resultant of many biological processes (biomarker measurement).

### Definition of biomarkers

In the current context, we will apply the concept of biomarkers as a way to characterize objectively nutrient intake and or nutritional status. There is a distinction between nutrient intake and nutritional status as exemplified in the case of vitamin D. The best way to evaluate vitamin D intake objectively would be to measure vitamin D_2_ and D_3_ in blood. However, the concentration of these vitamins is so low in blood that it is not feasible to measure these molecules by available technology. It turns out that a hydroxylated derivative of vitamin D (25-hydroxy vitamin D, formed in the liver) is a sensitive marker of how much vitamin D is found in the body based on the two main sources, diet and sun exposure over a period of months [6]. Another example of the distinction between intake and status can be polyunsaturated fatty acids (PUFAs). The best way to evaluate intake of especially PUFAs is to isolate the triglyceride-rich chylomicrons in the time interval of 2–6 h after the meal. Although it is difficult to monitor the amount of fatty acids consumed, it is possible to have good estimates on the quality of fatty acids by gas liquid chromatography (GLC) in combination with flame ionization detection (FID) [7]. The status of PUFA in the body is obtained best by having samples of tissues with slower turnover than plasma lipoproteins, like red blood cells, as can be obtained in whole blood samples and thus on dried blood spots (DBS).

The ideal biomarker is:

- Sensitive and specific for the nutrient or food it is supposed to monitor.
- Reflecting the period of interest concerning health or disease. Often in biomedicine, the long-term exposure is the most important.
- Unaffected by diseases or conditions of importance for metabolism of the actual nutrient. An example is plasma concentration of LDL-cholesterol, which is a risk factor for myocardial infarction, at the same time as a myocardial infarct by itself will reduce plasma LDL-cholesterol during the first days after infarction [8].
- Unaffected by other environmental or genetic factors. Often this is impossible to avoid, but the actual factors should be characterized and adjusted for.
- Inexpensive and reproducible to measure.

### Many variables influence biomarker concentrations

It is important to note that the concentrations of biomarkers in body tissues are influenced by many factors like digestion, absorption, distribution, transport, storage, metabolism, and export, as well as dietary characteristics like matrix differences, physical activity, the microbiome, environmental temperature, the use of drugs, and the presence of diseases. All these phenotypic, genetic, and environmental factors may give other results than what are obtained in traditional dietary studies based on 24 h recalls or food frequency questionnaires (FFQ). Thus, it is essential to be aware that in addition to provide objective data on nutrient intake or status, measurements of biomarkers represent many more biological processes than just food intake.

### Hypothesis-driven and data-driven search for biomarkers

The methods used to discover novel biomarkers can be divided into two categories: hypothesis-driven and data-driven [9]. Using the hypothesis-driven approach, prior knowledge might be obtained from food composition databases such as FooDB before methods are developed to measure the biomarker candidates of interest [9]. An example of the hypothesis-driven approach is the identification of pentadecanoic acid as a marker of dairy fat intake. Pentadecanoic acid is a saturated fatty acid with odd numbers of carbon atoms (15:0) and cannot be synthesized in the human body. However, pentadecanoic acid can be synthesized by the bacterial flora of the rumen of ruminants. Wolk et al. showed that the level of pentadecanoic acid in subcutaneous adipose tissue can serve as a marker of long-term milk fat intake [10]. Recently, the plasma phospholipid levels of pentadecanoic acid have been shown to associate with consumption of dairy fat and butter in adults [11]. The plasma or serum level of pentadecanoic acid represents a short-term marker for intake [12] and is inversely associated with type 2 diabetes [13]. The finding of a relationship between the plasma phospholipid levels of the trans-fatty acid elaidic acid (18:1, n-9) and intake of highly processed foods is another example of the hypothesis-driven approach [14]. Elaidic acid is generated during partial hydrogenation of vegetable oils and is used for the formulation of processed foods.

In the data-driven approach, there is no prior knowledge of the biomarkers. This makes the investigators measure as many lipids as possible, with the main limitation being the capacity of the analytical procedure. The recent study of Hanhineva et al. [15], studying the Nordic diet, is a good example of a successful data-driven approach. Using non-targeted LC-MS plasma metabolite profiling, in a randomized controlled trial with 106 participants assigned to three dietary interventions for 12 weeks, they identified several lipid species as potential biomarkers for fatty fish intake. The suggested biomarkers for fish intake included EPA, DHA, lysophosphatidylcholine (22:6 and 20:5), lysophosphatidyl-ethanolamine (22:6 and 20:5), and the furan fatty acid 3-carboxy-4-methyl-5-propyl-2-furanpropanoic acid (CMPF). CMPF was clearly changed and positively associated with increased consumption of fish, and using a stepwise linear regression model, they observed that plasma CMPF is an even stronger independent marker of fish intake than plasma EPA [15].

## DBS technology

With modern technology, it is possible to measure accurately thousands of metabolites, as well as nutrients, in small biological samples. It is also possible to monitor hormones, peptides, and proteins to enhance the quality of nutritional evaluation. We will describe the principles for metabolomics, including lipidomics, in addition to measurements of different nutrient-relevant proteins and minerals.

With enhanced sensitivity for measurements of very small amounts of nutrients and other molecules, it is obvious that we do not need large biological samples but can obtain high-quality measurements based on small samples (microliters) collected by non-professional subjects, who are able to follow simple instructions. This will allow a marked reduction in costs and will make it much easier to collect samples from thousands of subjects, e.g., in remote study-fields.

DBS sampling has been used to screen newborn babies for metabolic diseases for more than 50 years [16]. A spot of blood from a heel stick is applied on a filter paper and allowed to air dry. A circular punch (about 3 mm) is removed, eluted, and analyzed for metabolic markers. More than 50 separate analytes can be measured from a quarter of a blood spot [17, 18], mainly due to adoption within the last decades of the high sensitivity of liquid chromatography (LC) combined with mass spectrometry (MS) (http://vitas.no/services/dried-blood-spots).

### Advantages of DBS

As an important part of alternative sampling techniques, we will focus on DBS and similar alternatives because:

- Sampling can be performed by non-professionals following simple instructions.
- Sampling can be performed anywhere: in the field, classroom, gym, workplace, and before, during and after sports competitions.
- DBS is close to being non-invasive.
- DBS requires much less material (uL compared to mL) due to the improved sensitivity obtained with modern chromatography in combination with use of sensitive detectors like mass spectrometry, fluorescence, and flame ionization [19–21].
- Sampling is much cheaper than classical blood sampling and does not require participation of health professionals.
- Sample stability is often very good for DBS but has to be validated for each analyte.
- Transport of samples is easier, is less expensive, and represents minimum biohazard compared to classical blood/plasma samples.
- Samples are easy to store in tissue banks due to stability and small volume.
- There is often no need for laborious blood processing before analyses.

### Disadvantages of DBS

Due to the fact that the DBS technology is relatively new, much less experience is accumulated than for classical measurements in plasma or serum:

- Less information is available on metabolites, nutrients, and proteins in the whole blood compared to classical plasma/serum samples.
- Measurements of every new analyte collected by DBS have to be validated—i.e., accuracy, reference values, pathological values, and reproducibility should be established before the measurements can be fully interpretable.
- Whole blood is much more heterogeneous than plasma/serum, including all components of plasma in addition to platelets and several cell types.
- Some analytes are differentially distributed between plasma and blood cells. Potassium is a classical example of an intracellular mineral with 15–50-fold higher concentration in red blood cells than in plasma [22].
- Small volumes of blood are available making it difficult to measure analytes with very low blood concentrations.
- The quality of DBS can be poor because sample donors do not follow instructions concerning sampling, drying, and mailing.
- Some donors are hesitant to perform their own finger-prick sampling.
- The exact volume of blood might be difficult to obtain because the blood sample might be unevenly distributed on the filter paper.

### Applications of DBS

The DBS technology has been used for clinical and pre-clinical pharmacokinetic studies, taking advantage of smaller samples and simplified sample collection and handling. DBS sampling has also been used for disease surveillance in developing countries [23], at home, in the pharmacy, in the gym, in sports competitions, and in large epidemiological studies [24–28].

### Validation of DBS technology

The best way to obtain high-quality measurements is to be aware of the pitfalls in the procedure and carefully standardize all steps in the use of DBS technology [29].

### Blood sampling

Cleaning of the finger, earlobe, or heel can be done with soap and clean water before the sampling place is dried. The sampling place (finger or heel most often) should be warmed in hot water (~40 °C) to enhance the capillary blood-flow and make sampling easier. It is also important to increase the pulse pressure by standing up during blood sampling. A safety lancet in the form of a needle or blade is used to penetrate the skin with a depth of 1–2 mm thereby cutting one or several capillary blood vessel. The initial drop of blood is dried off with a clean gauze pad because it may be contaminated with interstitial fluid [30, 31]. The free dripping blood drops are applied on the filter paper and should not be squeezed out blood by milking movements to avoid tissue fluid and hemolysis. Clotting, layering, or supersaturating the filter should be prevented. The predefined circle on the filter should be homogenously and symmetrically filled and both sides of the card/paper must show the same red color. Samples indicating contamination or hemolysis or with insufficient volume collected are not suitable [18, 29, 32] depending on the type of analysis.

In every procedure, we depend on a device to cut into the skin a few millimeters to get access to capillaries. There are many protected types of lancets available, which are released when pressure is applied on the lancet resting on the skin, thus providing blood drops for sticks or filter papers. Proper application of blood to the filter paper requires care to reduce artifacts due to uneven sample coverage; touching the paper, too large or too small blood drop, or too much time between the blood drops can make a sample unsuitable for analysis. These difficulties may limit the ability of non-professionals to self-collect samples from home or a remote location. However, in a large internet-based dietary intervention study named Food4Me, it was possible to collect thousands of samples by and from non-professionals with acceptable quality provided the instructions were properly communicated [33].

### Sampling matrix

**Fig. 1:**
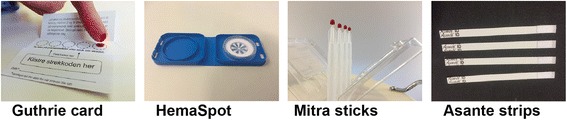
Different devices for collecting capillary blood samples in small quantities by non-professionals

The filter paper should be standardized in terms of particle retention, pore size, thickness, and weight (grams per square meter). Most filter papers are cellulose filters, and the recommended filters have a CE mark from the European Union (EU). Typically a 1.2-cm-diameter circle holds 30–100 uL per spot. It is important to collect enough blood to fill a circle in one go to obtain an even distribution of blood with an even thickness. One of the authors (TEG) has extensive experience with the standardized Whatman DBS filter paper 903 (Guthrie paper) from General Electric (GE). Since the 1960s and until recently, the only devices available have been different versions of the Guthrie card, a cellulose-based paper card with a clean and reproducible surface allowing even distribution of the blood on the paper. However, in the last 6–8 years, a number of new DBS devices have been developed. Some of these devices are shown in Fig. 1. The newly developed devices include different types of material like strips, sticks, and pens.

The Mitra sticks (Fig. 1) seem to be especially promising because they have a clearly defined volume of blood adsorbed on the gel matrix representing exactly 10.2 uL. The whole collected sample is used in analyses, without relying on punching out a representative area that could contain an amount of blood that is not as expected from the punched area. Another advantage is that it is easy to load the Mitra sticks into a machine for robotic handling of the whole analytical procedure. HemaSpot (Fig. 1) is a cartridge containing an absorbent paper and desiccant. Once blood drops are applied, the cartridge is closed and the desiccant rapidly dries the sample. Thus, long exposure to air during drying, with possible airborne contamination, is avoided.

Strips (Fig. 1) are designed to overcome non-specific binding limitations of classical dried blood spot cards; special low retention absorbing material is used, which in turn, readily releases proteins, enzymes, antibodies, DNA, and nutrient biomarkers. Strips may have an advantage because less samples may be wasted, and drying time is shorter than for classical DBS. In particular, strips have been used for sampling of blood for glucose measurements among subjects with diabetes [34]. Still, strips have for some reason been less used in recent times for new and more demanding analyses.

Pens can be used for finger-pricking, sample collection, and processing, and be integrated with commercially available paper-based assays [35]. This approach ensures safety and can be used by untrained end users in multiple settings. The pen format may provide low cost, high degree of safety, and robotization.

### Sample drying

Drying the samples in air is quite important to improve the stability of most metabolites, nutrients, and proteins. Most biological molecules are often stable under dry conditions. Required drying time is 1–4 h at room temperature of filters loaded with blood to make the analytes stable. Glucose can be metabolized in wet samples for weeks after collection of samples, whereas dry samples keep a much more stable glucose concentration. It is important to keep the samples dry also during transport (see below). Current methods require that the blood spotted onto filter papers should be dried in open air for a few hours prior to shipping or storage. This exposes the sample to potential contamination from circulating air and from foreign surfaces. Dried DBS samples in this manner may be stored at room temperature for many weeks, months, or years [36], depending on the analyte stability. However, samples containing unstable compounds should be stored at a lower temperature (≤−80 °C); [37, 38] to enhance the stability. Moreover, the drying rate can be variable based on ambient humidity; a sample will dry much more quickly in an arid atmosphere (e.g., Arizona) than a humid area (e.g., Amazon). Samples have greater stability with rapid drying and storage in low humidity conditions [20].

### Packaging, transport, and stability

Once the samples have been dried (usually 2 h at room temperature is sufficient), they should be placed in an airtight small aluminum envelope, with a small amount of dry silica to keep the humidity low during transportation. The small aluminum envelope can be placed in a regular postal envelope and sent by regular mail to the analytical laboratory for advanced analyses. Requirement for all analytes is that the stability should be evaluated during regular mailing/storage for up to 10 days, to make sure that the analyses are performed on high-quality samples.

### Sample extraction

For water-soluble analytes, the most common solvent is water, whereas different organic solvents like methanol, isopropanol, and chloroform are used for lipid extraction. For lipidomics of fatty acids, transmethylation is often performed in parallel with the extraction [26]. For extraction of peptides or proteins from DBS, enzyme-linked immunosorbent assays (ELISA) are mostly used with special buffers designed for optimal detection by the actual antibody, whereas a lysis buffer is used as a solvent when HbA1c is measured.

### Quality control

Many studies show that DBS sampling is compatible with, and equivalent to, current tests performed with fresh blood samples [39–43]. The accuracy and precision of a DBS LC-MS/MS method should be evaluated using quality control samples prepared at least at three different concentration levels (low, mid, and high), and analyzed along with a set of non-zero calibration standards in three separate validation runs. The lower limit of quantification (LLOQ) of samples must be assessed at least in one of the three validation batches. The intra- and inter-day accuracy, the bias (%) from the nominal concentration values, should be within ±15 % for all quality control samples except the LLOQs, for which a bias of within ±20 % is acceptable. The intra- and inter-day precision, assessed by the standard deviation divided by the mean coefficient of variation (CV%) from the replicate analyses, should be **≤**15 % for the results of all quality controls except the LLOQs, for which a **≤**20 % CV is considered acceptable [44].

Hematocrit is usually 0.41–0.51 for men and 0.37–0.47 for women [45]. The percentage might be out of the above ranges in certain populations, e.g., 0.28–0.67 for neonates (0–1 year old) and 0.35–0.42 for children (2–12 years old). Capillary blood tends to have a higher hematocrit (e.g., 0.61) than venous blood [46]. With high hematocrit, the viscosity of blood is enhanced and the diffusion of sample in the filter will be reduced, and the layer of blood will be thicker and the concentration of 31 amino acids and acylcarnitines was higher in the samples with highest hematocrit [47]. However, other studies do not show marked effect of hematocrit on 25-hydroxy vitamin D [48] or cyclosporine A [45]. Alternatively, MS signal for each lipid species can be standardized to the summed intensities of selected signals, providing a relative quantitation independent of blood volume and hematocrit level, as demonstrated by DBS studies of 3 months old infants compared with 12 months old children [49, 50].

The volume of the blood spot may also influence analytical results [44]. For every new analyte, there should be performed quality controls where the relationship between DBS area/weight and the amount of blood spotted on the paper should be examined by spotting increasing volumes of blood on the paper, and measuring the areas of the obtained spots [51], or weighing the obtained spots [52].

Chromatographic effects may also cause skewed distribution of blood and/or analytes on the filter paper. This is another factor that might cause significant differences in the measured analyte concentrations between central and peripheral areas within a spot. Different results have been reported depending on the analytes [47, 53]. During assay method development, it should be assessed whether the same analyte concentration could be measured from punches in different locations of the filter at different concentrations. Analyte classes with successful recovery and analysis from DBS include many metabolites (Table 1).

**Table 1:** Many types of metabolites, peptides, and proteins can be measured using DBS technology [29]. Examples of analytes measurable by the dried blood spot technique

Analytes class	Typical analytes
Small molecules	Amino acids, drugs, hormones, peptides, lipids, vitamins, minerals
Nucleic acids	DNA, miRNA, mRNA, RNA, virus
Proteins	Hemoglobin, cytokines, adipokines, myokines, thyroglobulin
Drugs	Anitepileptics, chloroquine, cyclosporin, gentamycin, paracetamol

DBS differs from blood plasma or serum samples mainly with respect to the presence of white and red blood cells. White blood cells make up only 1 % of the blood, whereas the red blood cells can vary between 30 and 70 % of the blood. The red color of blood is caused by the heme-iron complex and will interfere with many of the classical clinical chemistry methods using specific reagents in colored reactions detected by colorimetry. Thus, most analytical methods for DBS rely on separation of the analytes to be measured by means of chromatography, mass spectrometry, or antibody-based extraction. There is also a possibility of forming complexes with color that do not interfere with the heme complex or they may have fluorescent properties.

LC-MS/MS may be used for several types of analyses to measure the concentration of many metabolites like prostaglandins [28]. Solid-phase extraction (SPE) and liquid chromatography/tandem mass spectrometry (LC/MS/MS) may be used for the extraction, separation, and detection of 8-epi-PGF2α in DBS. Li and Tse [44] reviewed several aspects of DBS sampling in combination with LC-MS/MS, in particular focusing on lipid analyses and several lipophilic drugs.

In sandwich ELISA, a primary antibody is immobilized to the bottom of the sample container and the biomarker of interest is bound, whereas other constituents including the red color of heme are washed out. A secondary antibody is added that binds to the biomarkers and provides a chromophore, which can be measured by UV absorbance, fluorescence, or chemiluminescence. Many proteins, and some smaller molecules, can be measured by ELISA, which exhibits better specificity towards proteins than small metabolites like amino acids or drugs [54, 55].

Once a DBS sample has arrived in the laboratory for testing, a small punch (3–6-mm diameter) is taken from the card, eluted in a relevant solvent, and analyzed by a proper analytical method. The blood spot must be examined carefully to ensure that the sample punch is taken from a representative area. Uneven sample coverage due to poor application, variable hematocrit levels, or chromatographic effects may cause variable analytical results. Although the vast majority of analytical methods can be used with DBS, analyses requiring whole cells or volatile analytes are incompatible with DBS.

## Lipid profiling using classical lipidomics

Lipids include several classes of metabolites defined as substances extracted by organic solvents. They have several structural functions in cell membranes, lipid droplets, and lipoproteins [56]. Moreover, functional roles of lipids include membrane fluidity and microenvironment, signaling via eicosanoids and lipokines, ligands for transcription factors, and interaction with proteins based on hydrophobic as well as covalent bonds, and they often are important energy sources [5]. Whereas most lipids can be synthesized in the body, some fat-soluble vitamins and polyunsaturated fatty acids are essential, such as vitamin A, D, E, and K, and linoleic (omega-6) and alpha-linoleic acids (omega-3). These nutrients have to be obtained from the diet for mammals. One classification system divides the lipids into eight classes: fatty acyls, glycerolipids, glycerophospholipids, sphingolipids, sterol lipids, prenol lipids, saccharolipids, and polyketides [57]. Lipidomics represent the large-scale study of lipids present in a given cell, tissue, or organism at a defined time-point. It can be used to relate variation in lipid composition in biological samples to consumption of specific lipids, foods, or diets [4, 15, 58, 59].

### Dietary lipid biomarkers in tissues, plasma, and sera

A variety of tissues and plasma/sera specimens have been studied in search of biomarkers for intake of dietary lipids [60–62]. Adipose tissue and plasma are the most studied biological samples concerning biomarkers for dietary fatty acid intake, and they are considered the biological samples to choose for the study of relative intake of PUFA [63]. The composition of fatty acids in adipose tissue is to some extent determined by the habitual fatty acid intake [64, 65]. This is due to the slow turnover of fatty acids in the adipose tissue as well as in red blood cells. The half-life of fatty acids in adipose tissue is estimated to be between 6 months and 2 years [66–68]. Direct measurement of lipid age in subcutaneous fat using a ^14^C method, showed a mean lipid age of 1.6 years, which is consistent with a half-life of approximately 400 days [66–69]. Whereas the fatty acid composition of stored triglycerides is influenced by diet, the structural lipids in adipose tissue seem to be less influenced because of special functional requirements [64]. The fatty acid pattern of plasma phospholipids and cholesteryl esters is mostly reflecting the dietary intake of the past few weeks [70]. After 14 to 20 h of fasting, the plasma free fatty acids (also called non-esterified fatty acids) composition is dominated by the release of fatty acids from adipose tissue [71]. Thus, the free fatty acid composition of plasma from a fasting individual may serve as a surrogate for the fatty acid composition in adipose tissue. As an example, Leaf et al. [65] found correlation coefficients of 0.94 and 0.83 between adipose tissue and plasma phospholipid fractions of eicosapentaenoic acid (EPA) and docosahexaeonic acid (DHA), respectively. The red blood cells (RBC) may be a useful long-term marker of fatty acid intake, as the RBC turnover is 120 days [72]. Thus, when dietary information is collected to be compared with lipid composition in biological samples, the time frame must be considered [59].

### Exogenous fatty acids as biomarkers

Exogenous fatty acids (not de novo synthesized) serve as the best candidates for dietary biomarkers. Although biomarkers representing dietary intake of total fat and saturated fatty acid (SFA) have demonstrated conflicting results [73], PUFA and monounsaturated fatty acids (MUFA) measured in adipose tissue and plasma appear to be more valid [63]. The fact that the total pool of fatty acids in circulation represents both de novo synthesized (endogenous) and dietary (exogenous) fatty acids has made it difficult to find biomarkers for total fat intake [63]. However, one study showed that the combined changes of a group of fatty acids in RBC, plasma phospholipids, and cholesterol esters, in response to a low-fat or moderate-fat diet almost perfectly discerned between the total fat consumptions [72]. The same authors reported systematic increase in many endogenous fatty acids in response to a low-fat diet, despite reduced consumption of these fatty acids [72]. It has also been shown that high carbohydrate diets promote increased de novo synthesis of palmitic acid [74]. A recent study showed that the level of pentadeconic acid (15:0) in plasma and RBC reflected saturated fatty acid intake within an 8 weeks period [75]. Interestingly, there was no change in pentadeconic acid content in adipose tissue triglycerides [75]. Thus, future studies investigating changes in dietary intake of saturated fatty acids for up to 2 months might concentrate on plasma or RBC, as can be obtained in DBS.

### Dietary interventions and observational studies

The pattern of dietary PUFAs correlates with the fatty acid pattern in plasma and adipose tissue in dietary interventions as well as observational studies [58, 65, 76]. Already in 1966, Dayton and colleagues showed in a group of 393 institutionalized men, that increasing dietary intake of linoleic acid from 11 % to almost 40 % of total fatty acids, enhanced percentage of linoleic acid in serum as well as adipose tissue [58]. The content of linoleic acid in lipids from adipose tissue increased from 11 to 32 % after 5 years with the diet high in linoleic acid [58]. Supplementing the diet with marine n-3 fatty acids for more than 12 months caused enhanced incorporation of EPA and DHA into adipose tissue fatty acids [65]. In a study comparing Greenland Inuits and white Danes, it was shown that the Inuits had a higher concentration of EPA in plasma, probably reflecting their much higher consumption of very-long-chain n-3 [76]. Andersen and colleagues observed a correlation coefficient of 0.51 and 0.49 between dietary intake of EPA and DHA and corresponding plasma phospholipids, respectively [4]. Finally, the sum of EPA and DHA in RBC membranes is often called the Omega-3 Index. This index has been shown to discern between different dietary intake of EPA and DHA [77]. However, the omega-3 index might not be better than measuring EPA and DHA in plasma phospholipids or whole blood [77].

## Fatty acid profiles based on DBS

The search for efficient biomarkers has been hampered by the fact that most studies are relatively small scale. For the last 10 years, the lipid profiling assay developed by Marangoni and coworkers has been extensively tested in field studies [78]. The Marangoni-assay includes drying a blood drop on a filter paper strip containing the antioxidant butylated hydroxytoluene (BTH). The paper strip can be stored, then subjected to transmethylation (HCl and methanol) at high temperature, which will methylate fatty acids for GLC-MS. Large-scale cross-sectional studies with several thousand participants have been performed [79, 80], as have studies under field conditions in Cambodia and Tibet [81, 82] with several supplement interventions and validations (Table 2).

**Table 2:** Overview of studies measuring FA lipid profile by dried blood spot technique

Author	Subjects	Objective
Marangoni (2004) [78]	100 (46M, 54F)	Founder paper establishing method
Agostoni (2005) [91]	39 (22M, 17F) + 95 controls	Study infants of smoking mothers
Agostoni (2007) [81]	191 (100M, 91F) + 21 Italian controls	Intervention Cambodian infants (12 months)
Marangoni (2007a) [83]	10 (5M, 5F)	Walnut intervention (3 weeks)
Marangoni (2007b) [86]	108 (47M, 61F) - 10 (5M, 5F)	Cross-sectional study PUFA intervention (21 days)
Agostoni (2008) [90]	106 + 53 controls	Study infants of smoking mothers (follow-up of Agostini (2005) [89])
Risé (2008) [82]	13 (13M, 0F) + 14 Italian controls	Diet and FA profile study of Tibetians
Agostoni (2011)	16 pairs	Study of whole blood FA in infant, cord and mother
Saga (2012) [80]	3476 (1463M, 2013F)	Cross-sectional study of FA profile in Scandinavian population
Risé (2013) [79]	1835 total	Cross-sectional study of PUFA
	- 81 infants	
	- 728 children	
	- 434 adults	
	- 592 elderly	
Hinriksdottir (2015) [92]	52 (19M, 33F) + 25 controls	PUFA enriched fish meal intervention

Furthermore, method development has revealed and resolved several methodological challenges [39–42], providing a more robust method for future nutrient analyses. Recently, a breakthrough paper described DBS stabilization by chelators, which seemed to eliminate iron-promoted oxidation of PUFA, resulting in an excellent correlation (*r* = 0.97) between venous blood samples and DBS samples [43].

### Small scale nutritional studies by DBS

Four walnuts per day, containing 1.2 g ALA and 4.4 g LA, for 3 weeks, favorably affected the n-3 LC-PUFA status of volunteers (*n* = 10) [83]. Time course of measurements included 2 weeks run-in period and a 2 weeks washout period. However, the high EPA values are in contrast to two other studies with larger doses of walnuts [84, 85].

A cross-sectional study investigated the fatty acid profiles in a drop of blood from a fingertip and correlated with physiological, dietary, and lifestyle parameters in volunteers. A total of 108 healthy volunteers (47 males, 61 females), including 8 pregnant women, were questioned for dietary and lifestyle habits for the last 3 days and blood collected. In addition, 10 volunteers ingested either capsules (350 mg EPA, 300 mg DHA) or 200 g/week of smoked salmon, for 3 weeks [86]. These early studies indicated that the DBS method was suitable for cross-sectional studies as well as supplementation studies.

### Population screening by DBS

The Marangoni DBS technology was early used for screening fatty acid profiles under field conditions in less developed countries like Cambodia [81] and Tibet [82]. The effects of supplementation of two micronutrient powders on fatty acid status in Cambodian infants (*n* = 204) were compared in a 12 months intervention [81]. The fatty acid profiles of blood in a Tibetanian population (*n* = 13, Italian controls *n* = 14) were significantly correlated with dietary fatty acid patterns from the same population [82].

In a large cross-sectional study on blood from Italian infants, children, adults, and elderly, different patterns of n-6 and n-3 PUFA levels were observed. Data from four cohorts of Italians (*n* = 1835) were pooled in four age groups: 4 days old, 2–9 years old, adults (40–59 years), and elderly (60–79 ears) [79]. This study showed that the Marangoni-assay could be used in a large cross-sectional study. The study also showed that the DBS assay may allow detection of distinct PUFA profiles in new born infants. Large cross-sectional population studies in Norway and Sweden (*n* = 3476) have shown that food supplements like cod liver oil are in common use especially in middle-aged and older subjects, with marked influence on the fatty acid profiles [80].

### Ethics and lipid profiling of children

Samples of relatively small DBS from finger- or heel-pricks represent an important development due to the increased range of experiments that can be performed in an ethical way, like screening of infants [79, 87], very old patients [88] or disadvantaged, and cognitively challenged small school children [89]. Agostoni and coworkers reported a 24 % reduction in DHA (22:6) in children with mothers smoking throughout pregnancy (*n* = 159) [90]. The study was a follow-up of a smaller study observing the same effects (*n* = 19 smokers + 20 first trimester smokers + 95 reference controls) [91]. In a recent cross-sectional study 493 school children, aged 7–9 years, provided blood fatty acids obtained from finger-prick samples; the results showed that low blood n-3 PUFAs was associated with poor cognitive performance and behavior [89].

### Commercial applications of DBS

The ease and flexibility of sampling blood using DBS technology have revealed new commercial applications, such as demonstrating the bioavailability of long-chain n-3 PUFAs in fish oils. To fortify foods with PUFA from marine sources has remained problematic, due to the strong odor and taste. Hinriksdottir et al. added flavor-neutral microencapsulated marine fish oil to meals and compared with meals fortified with liquid fish oil and placebo control meals. Icelandic individuals (*n* = 99) were studied in a 4-week double-blinded dietary intervention in three groups [92], demonstrating that bioavailability of PUFA in encapsulated powder is very similar to meals enriched with liquid fish oil.

The supplement industry has also used DBS technology to demonstrate bioequivalence of different n-3 supplements [40, 93]. In the latter study, the n-3 fatty acid status of 50 young men was determined. In 10 individuals, the effect of supplements was investigated with time course from 2 to 24 h [93]. In times of increased competition for consumers and increased demands for new products, the demonstration of supplement efficacy might be an important competitive advantage.

### Cardiac disease studies using DBS

In a 3 g per day, PUFA supplement study of cardiac patients, using DBS technology, no effect on atrial fibrillation was seen after 6 months. The study was a randomized, double-blind, multicenter study including 204 Italian patients [94].

In another Italian study of patients with a recent myocardial infarction, matched case and control pairs (*n* = 112) showed that whole blood n-6 and n-3 PUFA levels were reduced. Using food frequency questionnaire (FFQ) demonstrated for 86 cases and 72 controls significant correlations between reported fatty acid intake and measured fatty acid pattern from DBS [95]. In contrast, another study applying DBS-based analyses showed no difference between fatty acid profiles of patients with arrhythmia without or with myocardial infarction and controls at hospital admission [96].

## Methodological challenges and refinement in lipid profiling using DBS technology

### High-throughput DBS analyses

DBS technology was originally used for screening of genetic diseases and mailing the samples to core laboratories [97, 98]. Stabilization of PUFAs by butyl hydroxyl toluene (BHT) has been successfully used [99]. Several researchers have increased the throughput of the fatty acid analyses using microwave oven transmethylation [100, 101]. Improved methodology allowed more frequent sampling and time series of fatty acid profiles in a fish oil supplementation study (*n* = 16) over 4 weeks and a washout period over 8 weeks [101]. In a study of soldiers (*n* = 287), fatty acid profiles were reported to be available 1 h after finger-prick sample collection [102].

### DBS method challenges and refinement

Some scientists have advocated BF_3_ use for transmethylation, but clear superior results using BF_3_ have not been demonstrated [100]. The less strict necessity of fasting blood samples is an important issue in self-administrated tests. Stark and coworkers [103] reported excellent stability of blood samples on paper immersed with BHT over 8 weeks at room temperature, although other scientists observed lower stability, in particular for DHA [41].

### The importance of chelators

Interestingly, Stark and coworkers [104] demonstrated a striking difference between DBS samples stored with or without BHT at −20 °C. In contrast, storage at room temperature, 4 and −78 °C showed little or no effect, which may suggest that ice crystal damage to membranes and release of chemicals within cells has important consequence for fatty acid stability. Metherel et al. in a follow-up study demonstrated that loss of PUFA probably was due to release of iron from heme in erythrocytes and advocated glycerol addition for freeze-protection of RBC [42].

A recent study reported that adding chelators (such as EDTA) to the DBS papers increased correlations between stored DBS samples and venous blood control samples markedly (*r* > 0.97) [43]. Similar improvement was also observed when ascorbic acid was added to DBS filters and during extraction, to improve the stability of vitamin A [105].

## DBS profiling of vitamins A and D

Vitamin A deficiency has long been recognized as a major cause for blindness among children in developing countries and to increased risk of infectious diseases [106, 107]. The use of DBS technology for population screening and monitoring of vitamin A status is often used in developing countries. The National Facility for DBS Technology for Vitamin A Estimation (Hyderabad, India) has carried out a vitamin A symptom study of 8777 pre-school children. A sub-group of 407 children with symptoms had DBS samples analyzed, finding vitamin A deficiency, particularly among rural children 3–5 years of age, and of lower socioeconomic class [108]. In a study in West Bengal, of 9228 children, 590 children had vitamin A deficiency, with higher incidence among boys than girls, and increasing deficiency with age [109].

A study in Guinea-Bissau of 1102 children (6–24 months of age), using DBS combined with ELISA for retinol-binding protein, observed a high prevalence of “vitamin A deficiency” (defined as RBP concentration equivalent to plasma retinol below 0.7 μmol/L) varying with season, ethnicity, and vaccination status [110]. A higher prevalence of vitamin A deficiency was found in children with infection, which is consistent with a study in Uganda of 661 children (6–59 months of age), demonstrating that infection status (measured by C reactive protein (CRP)) influenced the ELISA values for retinol-binding protein. Another interpretation is that dietary vitamin A deficiency causes reduced immune function.

Commercial offers for measuring vitamin A status are available [111–113]. However, there exists some controversy on the efficacy of sampling and extraction techniques [114]. A recent paper obtained a high correlation (*r* = 0.97) between venous blood and DBS samples from healthy subjects (*n* = 24) using acidic extraction [105]. Similar results were demonstrated 30 years ago, where the loss of vitamin A in serum samples was eliminated when adding ascorbic acid before extraction [115].

Vitamin D is linked to rickets, skeletal deformities, and bone disease. More recently vitamin D deficiency has been suggested to increase risk of many chronic diseases such as certain types of cancer, autoimmune diseases, cardiovascular diseases, and diabetes [116, 117], although intervention studies do not support the results based on observational, epidemiological studies [6].

An early study to optimize DBS technology for neonatal 25-hydroxy vitamin D status was performed by Eyles et al. [118, 119]. In a study of 118 archive samples stored up to 22 years, clear seasonal variations were detected but no annual variation, suggesting that DBS technology is reliable and promising for investigation of archive material [118]. In a follow-up study, neonatal cord serum and matched DBS samples (*n* = 100) were compared, finding them to be highly correlated (*r* = 0.85) [119].

Validity and reliability of the DBS technique was further investigated in plasma and matching DBS samples (*n* = 62) [120]. Commercial kits for DBS for vitamin D are available [121]. The feasibility of self-sampling of blood and saliva on filters was studied in a Norwegian breast cancer screening program (*n* = 381), reporting that postal service transport was efficient and low cost [24].

There are rather few studies using DBS in studies of older individuals [17]. Vitamin D status of seniors (> 60 years old, average age 72 years) was studied in 224 subjects in the ethnically diverse Older Adult Centre in Toronto [122]. No major differences between ethnic groups were found, although women had higher vitamin D status than men. Supplements were identified as the major factors responsible for the uniformly high vitamin D status. The concentration of 25-hydroxy vitamin D in blood has also been found to correlate negatively with cortical thinning in the brain during normal aging [26].

The vitamin D status in older adults in Toronto [122] contrasted starkly with a study of vitamin D status in young adults (*n* = 351), which showed that subjects with South Asian and East Asian ancestry had substantially lower 25-hydroxy vitamin D concentrations than subjects with European ancestry [123]. However, vitamin D status in 185 pairs of adolescent twins (average age 16 years) was found to be highly heritable (0.86) [124]. In a global perspective, the socioeconomic factors of vitamin D status evaluated by DBS sampling was emphasized in a cross-sectional study in rural Nepal, where 280 healthy children (12–60 months of age) were screened, reporting widespread (> 90 %) vitamin D deficiency [25].

## Water-soluble vitamins

### B-vitamins

DBS has been used quite successfully for measurements of folate [125], 5-methyltetrahydrofolic acid [126], as well as a sensitive marker of vitamin B_12_ deficiency, methyl malonic acid (MMA) [127]. Scolamiero et al. [128] screened 35,000 newborns over 6 years using DBS. Those showing altered propionyl carnitine (C3), 10 % of the subjects, underwent second-tier testing of MMA, finding 7 cases of acquired vitamin B12 deficiency. Algorithms, combining input for several analytes and genetic disease models, from very large data sets, have been developed. For example, Weisfeld-Adams et al. [129] reported on screening of 1,006,325 infants in New York from 2005 to 2008, in which 10 cases of confirmed cblC mutations causing vitamin B_12_ metabolism disorder were found. DBS data were retrospectively studied to validate the algorithm [129].

### C-vitamin

Vitamin C, or a range of carotenoids and flavonoids, has been widely used as general biomarkers of intake of fruit and vegetables [9, 130]. We have not found any studies using DBS to measure vitamin C.

## Amino acids, proteins, minerals, and trace elements

### Biomarkers of protein intake

An assessment of protein intake has been used extensively to determine nutritional status in subjects at risk of undernutrition, as well as among various patient groups, e.g., those with chronic renal disease, obesity, or in need of energy restrictions [131, 132]. The classical way to evaluate protein status has been to study nitrogen balance, in particular the urinary output of nitrogen. Bingham [133] reviewed the use of urine nitrogen as a biomarker for dietary protein intake and concluded that the method was reliable and inexpensive. However, it is a tedious and inaccurate procedure as it involves at least one, but preferably several, 24 h samplings of urine, and the study subjects should be in nitrogen balance. Moreover, measurement of urinary nitrogen tends to underestimate protein intake at high levels and overestimate at low protein intakes.

Measurements of single protein molecules have been used to assay whole protein intake and protein status. Among the most widely studied are prealbumin and albumin, which are produced endogenously by the liver. Recent data have discredited the use of albumin, in particular, as biomarker for protein intake, as it seems to be markedly affected by coexistent morbidities, especially in cancer and inflammatory disorders, as reviewed by Lee et al. [134]. Creatinine, creatine, and transferrin are other candidate biomarkers for protein intake, but clinical studies have not shown reproducible results [135, 136].

Meat is among the protein rich foods, and several biomarkers have been used to determine protein intake following meat consumption. Cross et al. [135] performed a randomized crossover study feeding 17 adults with various types of red meat for 15 days. Based on urinary excretion, they concluded that 1-methylhistidine and 3-methylhistidine were good biomarkers, which is in line with the reviews provided by Dragsted [136] and Scalbert et al. [9].

Petzke and Lemke [137] used a different approach to estimate protein intake, namely by determining hair isotope compositions. They studied if additional meat intake (200 g pork fillet/day) or omission of meat and meat products had an impact on ^15^N and ^13^C within 4 weeks in hair and plasma of young women. They concluded that hair protein ^15^N and ^13^C abundances take more than 4 weeks to show animal protein intake, in these women with a habitual daily protein consumption of more than 1 g per kg body weight. Stable isotope ratio analysis at natural abundance in human hair protein offers a non-invasive method to reveal information about long-term nutritional exposure to specific nutrients, including proteins [137]. However, the use of isotopes in hair as biomarkers of protein intake requires more testing, in particular in randomized intervention studies.

### Biomarkers of amino acid intake

Among the about 100 amino acids found in nature, 20 of them serve as building blocks and metabolites used for energy and in signaling pathways in humans, 8 of which are essential and have to be supplied in the diet. Traditionally blood and urinary concentrations of amino acids have been used as biomarkers of their intake. Recent methodological advances have also made it possible to assess local amino acid contents in hair [138] and locally in various organs, e.g., in neuronal tissues [139]. The advent of DBS to collect and store blood samples has opened opportunities to assess biomarkers in vulnerable populations, like premature infants and in populations in developing countries where access to freezers is limited. High-performance liquid chromatography and tandem mass spectrometry can be used to assess the amino acid concentrations from DBS and with satisfactory results [140].

Historically, the importance of amino acids has mostly been related to disorders due to deficiencies in amino acid metabolism such as maple syrup disease and phenylketonuria. However, there is increased focus on the use of amino acids including the claimed benefit of branched amino acids (leucine and isoleucine) to enhance physical performance [141], as a risk factor for cardiovascular disease (l-arginine) [142], and to improve cognitive function (tyrosine) [143]. Given the multitude of functions amino acids, more studies are warranted to delineate how well blood concentrations of amino acids and urine reflect subcellular levels of different amino acids.

### Iron

Iron is part of the heme molecule, which is an integral component of hemoglobin. In addition, iron is an important constituent of enzymes such as in the mitochondrial respiratory chain (cytochromes). According to the World Health Organization iron deficiency anemia still ranks among the top 5 causes of years lost to disability globally (http://www.who.int/maternal_child_adolescent/epidemiology/adolescence/en/) and continues to be a problem among adolescent girls living in developed regions [144].

The body stores of iron can be used as a proxy of long-term iron intake and can be determined in various ways. The classical way of estimating body iron content is the assessment of iron in bone marrow using light microscopy, although this approach yields only semi-quantitative estimates. To evaluate iron overloading, in particular in patients receiving frequent blood transfusions, imaging techniques like magnetic resonance imaging (MRI) have been used [145]. However, these are tedious procedures, and hence blood biomarkers are much more frequently used. Serum levels of ferritin are in most cases a reliable estimate of body iron stores, although it is affected by a range of concomitant disorders, in particular inflammations. Serum levels of ferritin also increase with age. Complementary to ferritin is the ratio between serum levels of iron and its transport protein transferrin, denoted iron-, or transferrin saturation. This is also affected by individual health status and pregnancy. It is debated whether serum ferritin levels and transferrin saturation levels correlate [146]. To circumvent these pitfalls, the use of serum levels of soluble transferrin receptor has gained increasing attention, as this is mostly independent of coexistent disease. Cook et al. [147] found excellent correlation between whole body iron content (measured according to known ferritin and hemoglobin levels after phlebotomy in healthy subjects), and the ratio of soluble transferrin receptor to ferritin; the latter two biomarkers being measured by ELISA.

Ferritin as well as transferrin receptor can also be measured using DBS with accuracy comparable to whole plasma values [148]; this facilitates measurements of iron status in remote areas where anemia is frequent. For example, the use of DBS to measure soluble transferrin receptor and hemoglobin was successfully applied among pastoral women of fertile age residing in rural North-Kenya [149].

### Selenium

This trace element is mostly found in enzymes involved in the human antioxidant defense system. Deficiency of selenium has been linked to many conditions including cardiovascular diseases and different forms of cancer. Many grain-based foods contain selenium, although its availability depends on the concentration of selenium in the soil. Vacchina et al. [150] recently described a method to assess selenium using mass spectrometry following acidic digestion of the DBS. In addition to plasma selenium, selenoprotein 1 and glutathione peroxidase activity are responsive to changes in selenium intake. However, their use as biomarkers for selenium intake is limited by inconsistent response to selenium intake [151] and might be explained in part by ethnic differences [152]. Moreover, Ashton et al. [151] concluded that there was insufficient evidence to assess the usefulness of other biomarkers of selenium status, including urinary selenium, plasma triiodothyroxine/thyroxine ratio, plasma thyroxine, plasma total homocysteine, hair and toenail selenium, erythrocyte, and muscle glutathione peroxidase activity. Currently, no biomarker is available reliably mirroring (i) variable selenium intake and (ii) selenium intake in different subpopulations.

### Zinc

Zinc exerts several functions, including stabilization of membranes, co-factor of transcription proteins, and as part of metalloproteinases. The plasma/serum levels of zinc and the erythrocyte zinc content have traditionally been the most common ways to evaluate zinc intake. The WHO report from 2007 on intake required to prevent zinc deficiency recommend serum levels of zinc as a biochemical marker of zinc status [153]. However, results are conflicting regarding their sensitivity to low and high zinc intake [154]; thus, new biomarkers for zinc intake are needed. Recently, Reed et al. [155] used a chicken model (*Gallus gallus*) to propose the erythrocyte linoleic acid/dihomo-γ-linolenic acid ratio as a sensitive biomarker of alterations in zinc intake. This was based on previous findings that this broiler chicken is sensitive to dietary intake of zinc [156]. In addition, a similar membrane fatty acid composition has been reported in mammals, which makes it possible to take advantage of a link between the ratio of these two essential fatty acids and mineral intake to evaluate zinc status [157]. The DBS method developed by Vacchina et al. [150] can also be used to assess zinc.

### Magnesium

Magnesium is an important component of bone and plays a role in energy metabolism and protein and nucleic acid synthesis and is a co-factor for many proteins and hormones. Magnesium is mostly located intracellularly in spite of the fact that measurements of serum and urine levels of magnesium are usually performed to evaluate magnesium intake. Notably, ethnic variations and concomitant intake of other trace elements like sodium and calcium may affect these measurements [158]. Another less studied biomarker of magnesium intake is the content of magnesium in erythrocytes [159]. Witkowski et al. [160] performed a systematic review of analyses of 20 biomarkers of magnesium intake and concluded that the serum or plasma magnesium concentrations, erythrocyte concentration, and urinary magnesium excretion responded to dietary manipulation and could be used as biomarkers. We have not been able to find any scientific articles published concerning measurement of magnesium applying DBS technology although preliminary data show that whole blood analyses are feasible.

### Chromium

Chromium is important for the metabolism of glucose, protein, and lipid by virtue of its action as co-factor for several enzymes. To evaluate exposure to chromium in foods and liquid intakes, measurements of both plasma and hair have been used. Sazakli et al. [161] performed a population-based cross-sectional study of chromium exposure and intake in Greece, a country with higher than WHO-recommended levels of chromium in the drinking water. Both the plasma concentrations and the hair levels of chromium were associated with intake in different Greek regions. Urinary chromium may not be a valid biomarker for chromium intake [162]. There is a need for better biomarkers of chromium intake [163]. We have not been able to find any scientific articles published concerning measurement of chromium applying DBS technology although it should be feasible.

### Fluoride

Fluoride is associated with dental enamel and bone density. Little is known about useful biomarkers of assessing fluoride intake. Rugg-Gunn et al. [164] concluded in their review that: “While fluoride concentrations in plasma, saliva, and urine have some ability to predict fluoride exposure, present data are insufficient to recommend utilizing fluoride concentrations in these body fluids as biomarkers of contemporary fluoride exposure for individuals. Daily fluoride excretion in urine can be considered a useful biomarker of contemporary fluoride exposure for groups of people.” We have not been able to find any scientific articles published concerning measurement of fluoride applying DBS technology although it should be feasible.

### Mercury

Measuring mercury intake is important to control for the toxic effects of this trace element, in particular concerning the developing nervous system. The concentration of mercury in plasma as well as its content in hair has been used as biomarkers for mercury exposure, and they are apparently well inter-correlated [165]. Blood samples can be assayed using DBS [166]. The urinary excretion of mercury offers some promise as a biomarker of mercury intake [167].

### Cadmium

Similar to mercury, undesirably high intakes of cadmium may lead to toxic effects. Often the plasma concentration (e.g., as in DBS) is used as a biomarker of cadmium intake [150, 166]. Interestingly, Piasek et al. [168] reported a potential usefulness of cadmium content in the placenta for evaluating cadmium exposure during pregnancy.

### Iodine

Iodine is essential for adequate thyroid function. To assess iodine intake, direct measurements of urine iodine as well as of iodine incorporated into thyroid-derived molecules (e.g., thyroxine, thyroid-stimulating-hormone and thyroglobulin) have been used as functional markers [169]. The systematic meta-analysis by Ristic-Medic et al. [169] supported the use of all of these biomarkers for evaluating iodine intake, although to a varying degree, urine excretion being the better [170]. Moreover, the analysis of thyroglobulin in DBS has emerged as a putative biomarker alternative for iodine intake [171].

## Feces—a potential matrix for dietary biomarkers

It has been generally acknowledged that the gut microbial ecosystem may influence human physiology and health [172]. The understanding to what extent the intestinal microbic composition is subject to dietary control, and to integrate these data with functional metabolic signatures and biomarkers is of utmost interest [173]. The gut microbiota can be recognized as a highly active metabolic organ because it affords metabolites affecting physiological processes in the intestine and beyond. Thus, gut microbiome metabolites interfere with the metabolic phenotype of the host and consequently may affect health and disease risk [174].

### Diet and stool metabolites

Diet plays a pivotal role in shaping the human gut microbiota (composition and metabolism), one of the most densely populated microbial ecosystems in nature. As a prominent example, prebiotics are used to modulate composition, metabolism, and function of the gut microbiota to improve the gut and host health [175]. However, a number of additional factors, such as physicochemical food properties, nutrient availability, colonic transit time, and age of the host, may modulate the effect of diet on the composition and metabolic activity of the colonic microbiota [172, 173]. Metabolites due to bacterial energy metabolism may reflect dietary intake, such as short chain fatty acids (SCFA) as a result of carbohydrate metabolism, metabolites of fatty acids and lipid bioconversion, and metabolites of protein fermentation. Minor food constituents structurally modified by microbial activity might be detectable in feces and could be a characteristic for the consumption of certain foods, especially plant foods (bioconversion of secondary plant products). Thus, the hypothesis that metabolites detectable in fecal samples may reflect dietary intake is valid, although not well elaborated yet.

### Short chain fatty acids

Carbohydrates that are not digested in the small intestine are fermented by colonic microbiota and produce SCFA, e.g., butyrate, propionate, acetate, and longer-chain fatty acids [176, 177]. The SCFA fecal concentration alters in different stages of life; e.g., the change from breast-feeding to solid food or a higher butyrate production at higher age as a consequence of an increase in *bacteroides* [178]. Furthermore, the relative proportions of SCFA differ from one person to another and they are specifically sensitive to the type of fermented carbohydrate [179, 180]. However, the current evidence for a distinct dietary substrate identification based on SCFA analyses in feces is not conclusive [172]. Also, lactate and succinate are intermediate metabolites of bacterial carbohydrate metabolism. However, a direct link to specific dietary carbohydrates has not been established; rather, they may be useful markers of gut health [172, 181].

### Prebiotics

Many studies administering prebiotics as inulin, oligofructose, and fructooligosaccharides (FOS) have reached a significant increase in fecal *bifidobacteria* counts [182]. A double-blind placebo-controlled crossover study has shown that the numbers of fecal *bifidobacteria* and *lactobacilli* have significantly increased after administering very-long-chain inulin (VLI), derived from globe artichoke, as compared to placebo [183]. Also oral administration of a similar dose (similar to inulin dose) of acacia gum increased *bifidobacteria* and *lactobacilli* production [184]. Thus, an increase in fecal *bifidobacteria* and *lactobacilli* excretion reflect prebiotics intake. Another study has shown that administering 10, 15, or 20 g/day of a sugar-free digestion-resistant dextrin for 20 days led to increased number in the fecal *lactobacilli* and *bifidobacteria* and a decrease in *clostridium perfringens* [185]. However, it should be considered that there are different methods and no standard protocols for assessing microbial proportions or numbers or activity in fecal samples [186].

### Branched chain fatty acids

Branched chain fatty acids (BCFA), ammonia, amines, phenols, cresols, indoles, hydrogen sulfides (highly volatile), etc. are metabolites arising from protein fermentation [187]. There is a considerable inter-individual variation in the urinary excretion of p-cresol and phenols probably reflecting its production in colon [172]. In addition, fecal concentrations of isobutyrate, 2-methylbutyrate and isovalerate, metabolites of the bacterial fermentation of valine, isoleucine, and leucine [188] decreased after intake of prebiotics [189–191]. Thus, such metabolites could be evaluated for its use as biomarkers of dietary intake. So far, they are used only as more general markers for bacterial protein fermentation [192].

### Dietary polyphenols

Most dietary polyphenols (e.g., flavonoids, anthocyanins, phenolic acids, stilbenes, lignans, tannins) pass the small intestine without absorption. Polyphenols have been described to modulate composition of the gut microbiome and probably contribute to gut and host health [193, 194]. In addition, polyphenols are bio-transformed into derivatives that may become bioavailable for the host. Studies using metabolomics techniques have shown that numerous microbial metabolites of polyphenols can be detected in feces and hence may be key markers for colonic bacterial composition and activity [195]. They may also be valid markers of food intake. As an example, serum concentrations of enterolactone and equol are measured as markers of plant lignan intake and microbial metabolism of daidzein (mainly derived from soy food), respectively. Using feces as the analytic matrix, the chances to get more information about diet (and microbial activity) would be high.

### Sterols

Secretions from the gastrointestinal tract constitute a substantial portion of feces, and the bile is a major contributor. Bile acids, cholesterol, coprostanol, and their metabolites are subject to the enterohepatic circulation. Their content and proportions (e.g., primary versus secondary bile acids) are strongly influenced by dietary fat (saturated fat) intake, concomitant carbohydrate intake and the gut microbial composition [196].

Fiber and resistant starch supplementation or changing to a lacto-vegetarian diet resulted in decreased fecal bile acid concentrations, particularly secondary bile acids [197, 198]. Cholesterol is transformed to coprostanol, which represents about 60 % of the sterol content and is considered the major 5ß-stanol in human feces [199]. Plant phytosterols, e.g., campesterol and sitosterol, are reduced by enteric bacteria into 5ß-stigmastanol and 5-ß campestanol. Hence, 5ß-stigmastanol and 5-ß campestanol may be used as fecal biomarkers for dietary phytosterol intake [200]. The relative proportions of animal- and plant-derived stanols in feces may reflect dietary preferences [200, 201]. Thus, the possibility of applying various metabolites of bile acids and steroids in feces as biomarkers of current diet should be further explored.

### Provitamin A carotenoids

Non-absorbed provitamin A carotenoids are mainly excreted in feces. In addition, absorbed provitamin A carotenoids are partially excreted through bile and pancreatic secretions in feces. Thus, carotenoids can be used as fecal biomarkers for provitamin A (e.g., ß-carotene) intake or for estimating ß-carotene bioavailability or for establishing ß-carotene net balance in the body [202]; e.g., Van Lieshout et al. [202] estimated the difference in the bioavailability of ß-carotene between pumpkin and spinach based on measuring carotenoids concentration in feces and serum.

### Future prospect

The use of metabolomics techniques for analyses of fecal samples allows identification of new biomarkers of gut health, as well as understanding the interrelationship between the human gut microbiome activity and host metabolism [203]. Moreover, it provides the possibility to establish new markers of dietary (food) intake. A successful strategy might be to utilize valid and reproducible metabolomics data to discover metabolite patterns in feces that are associated with diet (rather than looking for single compounds only). Thus, dietary intervention studies may allow identification of fecal metabolite pattern, which might be reproduced in population-based studies (e.g., cross-sectional and cohort studies). Up to now, only some intervention studies have used metabolomics techniques to evaluate the effect of synbiotics in humans [204–206].

### Methodological aspects

Metabolite profiling in fecal samples is much more complicated as compared to other biospecimen. The physicochemical properties of the feces influence the reproducibility and full coverage of metabolite profiling attained [206]. However, lyophilized feces samples extracted by water methanol mixtures, allow for the analysis of metabolite profiles that are reproducible and are composed of various compounds. There are different options for fecal sample collection and storage conditions that may impact on the metabolite concentrations, e.g., immediate freezing versus cooling on ice or in the refrigerator before freezing versus storage at room temperature before freezing [207]. For example, in water extracts from frozen fecal samples, the concentration of amino acids and glucose is higher than that in water extracts of fresh fecal samples [208]. Another option is immediate mixing of the collected fecal sample with stabilizing solutions such as RNAlater or Amis Transport Medium [209]. A requirement for use of fecal samples for dietary biomarker identification is a high sample quality; however, optimization and harmonization of sample collection, storage, and processing procedures are yet to be established.

Analytical tools such as NMR, GC-MS, LC-MS, and LC-MS-MS have been applied as metabolomics techniques for the analyses of stool metabolites. Up till now, mass spectrometry techniques have some advantages in characterizing human metabolomes due to their high sensitivity and selectivity [210]. NMR spectroscopy is widely used in metabolite profiling due to its non-destructive sample handling and its ability to quantify compounds at very low concentrations. Furthermore, NMR spectroscopy gives information on the structure of the compounds, which is useful when unknown compounds have to be identified [211, 212]. The heterogeneity of dietary compounds and of their formed metabolites after ingestion is still a big challenge, as many of these compounds are not yet defined [213]. Due to the diversity of fecal metabolites, more than one method is necessary to achieve a comprehensive metabolite profile [214]. Important measures for selecting an appropriate analytical method have to be considered such as coverage, dynamic range, selectivity, accuracy, precision, and price per sample [210].

## Conclusions

Biomarkers of nutrient intake or nutrient status are important objective measures of one of the most important environmental factors people are exposed to, namely food. It is very difficult to obtain accurate data on individual food intake, and there is a large variation in nutrient composition of foods. This is the background for studying more objective biomarkers of nutrient intake. Modern technology with high sensitivity and specificity concerning many nutrient-relevant biomarkers has allowed an interesting development of non-professional collection of small amounts of blood by finger-pricking and collection on filters or sticks. With proper collection, drying, transport, extraction, and analysis of the samples, many analytes of nutritional interest can be measured such as metabolites, lipids, vitamins, minerals, and many types of peptides and proteins. The advantage of this alternative sampling technology is that non-professionals can collect, dry, and mail the samples; the samples can often be stored at room temperature in a dry atmosphere; small amounts of blood are required for analyses in professional laboratories with modern analytical methodology. However, it should be noticed that chemical measurements of nutrient biomarkers are hampered by many confounding factors like variation in food matrices, difference in digestion, absorption, transport, distribution, activation, and catabolism. These facts make it obvious that we do not get comparable data from personal registration and objective biomarker measurements. Thus, food registration as well as biomarker measurements will most likely complement each other in future decades of nutritional sciences. Another promising area of potential biological interest is the biology of the microbiome in association with biomarkers. Interesting perspectives are also related to the marked trend of self-monitoring of body functions linked to mobile phone technology.
